# Responses of Plant Community Composition and Biomass Production to Warming and Nitrogen Deposition in a Temperate Meadow Ecosystem

**DOI:** 10.1371/journal.pone.0123160

**Published:** 2015-04-13

**Authors:** Tao Zhang, Rui Guo, Song Gao, Jixun Guo, Wei Sun

**Affiliations:** 1 Institute of Grassland Sciences, Northeast Normal University, Key Laboratory of Vegetation Ecology, Ministry of Education, Changchun, China; 2 Institute of Environment and Sustainable Development in Agriculture, Chinese Academy of Agricultural Sciences, Key Laboratory of Dryland Agriculture, Ministry of Agriculture, Beijing, China; 3 State Key Laboratory of Desert and Oasis Ecology, Xinjiang Institute of Ecology and Geography, Chinese Academy of Sciences, Urumqi, China; DOE Pacific Northwest National Laboratory, UNITED STATES

## Abstract

Climate change has profound influences on plant community composition and ecosystem functions. However, its effects on plant community composition and biomass production are not well understood. A four-year field experiment was conducted to examine the effects of warming, nitrogen (N) addition, and their interactions on plant community composition and biomass production in a temperate meadow ecosystem in northeast China. Experimental warming had no significant effect on plant species richness, evenness, and diversity, while N addition highly reduced the species richness and diversity. Warming tended to reduce the importance value of graminoid species but increased the value of forbs, while N addition had the opposite effect. Warming tended to increase the belowground biomass, but had an opposite tendency to decrease the aboveground biomass. The influences of warming on aboveground production were dependent upon precipitation. Experimental warming had little effect on aboveground biomass in the years with higher precipitation, but significantly suppressed aboveground biomass in dry years. Our results suggest that warming had indirect effects on plant production via its effect on the water availability. Nitrogen addition significantly increased above- and below-ground production, suggesting that N is one of the most important limiting factors determining plant productivity in the studied meadow steppe. Significant interactive effects of warming plus N addition on belowground biomass were also detected. Our observations revealed that environmental changes (warming and N deposition) play significant roles in regulating plant community composition and biomass production in temperate meadow steppe ecosystem in northeast China.

## Introduction

The global surface temperature has increased at a rate of 0.2°C per decade over the past 30 years as a result of rising greenhouse gas emissions [1], and global warming is expected to increase continuously for the next 100 years [2], which will severely affect terrestrial ecosystems. A number of studies have reported that one of the consequences of global warming on terrestrial plant ecosystem stability is significantly decreased species richness and diversity [3–5], but other studies have reported that warming had no effect on plant diversity [6]. Studies have found that functional groups have varying responses to warming [7] as well as a profound influence on plant productivity [8–9]. Furthermore, certain studies, through non-intrusive field experiments, have demonstrated that plant responses to warming are ecosystem dependent, with plants in cold-wet northern sites more sensitive to warming [10], while warming in other ecosystems has been reported to decrease the productivity in terms of both above- and below-ground biomass [11–12].

The increase in atmospheric nitrogen (N) deposition induced by human activities has been recognized as another important threat to terrestrial ecosystem that causes shifts in the plant community structure [13]. A large number of studies have found that N deposition in soil highly reduces plant diversity and species richness [14–17]. A number of studies, however, have demonstrated that N deposition does not actually changes the species richness of the vegetation [18–19] but rather increases plant diversity [20]. However, the ecological impacts of even a relatively small amounts of N deposition on plant species interactions at the species level are still not well understood [21]. In addition, nitrogen availability plays a more important role in limiting plant primary productivity than other available soil nutrients, and nitrogen deficiency is globally distributed [22–24]. In general, the response of grassland productivity to N deposition is determined by the degree of N saturation of a soil. Small amounts of N deposition can improve plant productivity before the soil N reaches the saturation point [8,22]. On the other hand, N deposition can also reduce plant productivity when the soil has reached the N saturation point [25].

Although amounts of studies had discussed the influence of warming or N deposition on plant community composition and productivity alone, warming and N deposition frequently occur simultaneously. Several previous studies have documented the existence of the additive effects of warming and N deposition on grassland diversity [6] and productivity [26–27] in North America. Beyond these studies, additional studies that have focused on the influence of warming and increased nitrogen deposition on ecosystem community composition and productivity are rare. Songnen grassland is located at the east edge of the Eurasian grassland belt, which is the most typical and the largest meadow steppe in China. Moreover, Songnen grassland belongs to the areas with highest soda saline concentration, and salinization is a very serious issue. It is predicted that the temperature will elevate by 2.8–7.5°C in the next 100 years in Songnen grassland in northeast China [2], which is one of the areas most intensively affected by nitrogen deposition as well [28]. Although, a number of previous studies have focused on the effects of warming and N addition on soil nutrient cycling [29] and ecosystem carbon fluxes [30] in Songnen grassland ecosystem, the influences of warming and N addition on plant community composition and productivity remains unclear. To ascertain the potential effects of climate warming and increased N deposition on the plant community composition and biomass production of Songnen grasslands, we conducted a field experiment with manipulated warming and N addition. In this experiment, we have aimed to answer the following questions. (1) How do warming and N addition affect plant community composition and biomass production in a temperate meadow ecosystem? (2) What are the influences of abiotic factors (e.g., soil moisture) and biotic factors (e.g., plant interspecific interactions) on plant community composition and plant biomass under conditions of climate warming and N addition?

## Materials and Methods

### Ethics statement

No specific permissions were required for the described field studies because the Songnen Grassland Ecological Research Station is a department of Northeast Normal University, and the performance of this study has followed the guidelines set by Northeast Normal University. No endangered or protected species were involved in this field investigation.

### Study site

The experiment was conducted at the Songnen Grassland Ecological Research Station (44°45′N, 123°45′E), Northeast Normal University, Jilin Province, China. The mean annual precipitation is approximately 400 mm, with 90% occurring from May to October. The mean annual average air temperature is 4.9°C, and the mean annual average land surface temperature is 6.2°C. The soil in the studied area is a soda-saline type soil, with a pH of 8.2 and 3–4% organic matter in the surface layer. The vegetation in the experimental site is dominated by *Leymus chinensis*, *Kalimeris integrifolia*, *Carex duriuscula* and *Phragmites australis*. The rate of natural restoration of the degraded vegetation is very slow.

### Experimental design

We used a complete randomized block factorial experimental design with two factors: warming and N addition. There were four treatments: control (C), warming (W), N addition (N), and warming plus N addition (W+N), replicated 6 times. The size of each plot was 2 m × 3 m. All of the warmed plots were heated continuously using infrared radiators (MSR-2420, Kalglo Electronics Inc. Bethlehem, PA, USA) suspended at a height of 2.25 m over the plot center. The infrared radiator radiates infrared heat waves that are identical to the sun's energy except for the absence of UV tanning rays. Each 165 cm × 15 cm infrared heater had a radiation output of approximately 100 watts m^-2^ [31], and the effects of the infrared heaters on soil temperature were spatially uniform [32]. In each control or N addition plot, one ‘dummy’ heater of the same shape and size was installed to mimic the shading effects of the infrared radiator. All of the heaters under the warming treatments were set to a radiation output of approximately 1700 W. Anthropogenic N deposition has been estimated at 80–90 g m^-2^ yr^-1^, and even higher N deposition is predicted to occur in the future as the result of land-use change and activities [33–34]. In northern temperate grassland ecosystems, the community saturation rate for N deposition is approximately 10.5 g m^-2^ yr^-1^ [35], whereas the N deposition has only been 2.7 g m^-2^ yr ^-1^ over the last decade in our area [36]. Thus, in the N addition treatments plots, ammonium nitrate (10 g m^-2^ yr^-1^) was applied as an aqueous solution on the first day in May annually. In the control and warming plots, the equivalent amount of water (without N) as for the N addition treatment was added to account for N addition-induced changes in the water availability. The experiment was started in May 2006 and was terminated in September 2009.

### Meteorological data collection

The mean monthly temperature and precipitation from 2006 to 2009 were recorded using an eddy covariance system installed 200 m away from the experimental site. One EM50/R probe (Decagon Ltd, Pullman WA, USA) was buried 0–15 cm from the soil surface in each experimental plot, measuring soil temperature (ST) and soil moisture (SM) at one-hour intervals.

### Plant diversity and plant biomass

During the growing season (in mid-August every year), the cover and frequency of each plant species was estimated using a modified point-frame method [37]. A frame containing ten 1-m long vertical pins arranged in parallel at a spacing of 10 cm × 10 cm was systematically placed within the central 1 m^2^ of the plot. The number of plant species, plant height and density present in the quadrat were recorded. The plant species occurred in experiment plots were showed in Table 1. The plant numbers per species were also used to calculate species richness (species number per quadrat), importance value (IV), diversity (Shannon-Wiener index *H*) and evenness value (Pielou index *E*).

$$Shannon-Wiener\;index\;(H):H=-\sum_{i=1}^{S}PilnPi$$ (1)

$$Pielou\;index\;(E):E=\frac{H}{lnS}$$ (2)

Where S is the total number of species and *Pi* is the proportional abundance of species *i* in total species. The importance values per species were calculated using the following formula.

Importance value: (IV) = (RC + RF + RD) /3 (3)

Where RC is the relative cover (RC = cover of one species / total cover of all species × 100), RF is the relative frequency (RF = number of quadrats in which the species occurred / total number of quadrats studied × 100), and RD is the relative density (RD = number of individuals of a species in all quadrats / total number of quadrats × 100).

**Table 1:** List of species within the experiment quadrats (1 m × 1 m) from 2006 to 2009.

Species		LHT	Abbreviation	Coverage (%)
Graminoid species	*Leymus chinensis*	P	Lc	62
	*Phragmites australis*	P	Pa	10
	*Setaria viridis*	A	Sv	2
Nongraminous forbs	*Limonium bicolor*	P	Lb	
	*Thalictrum simplex*	A	Ts	
	*Kalimeris integrtifolia*	P	Ki	13
	*Carex duriuscula*	P	Cd	5
	*Artemisia mongolica*	P	Am	
	*Artemisia anethifolia*	AB	Aa	3
	*Artemisia scoparia*	AB	As	
	*Inula japonica*	P	Ij	
	*Potentilla flagellaris*	P	Pf	
	*Polygonum sibiricum*	P	Ps	
	*Pocockia ruthenica*	P	Pr	
	*Xanthium strumarium*	A	Xs	
	*Lespedeza davurica*	P	Ld	2
	*Cynanchum chinense*	P	Ch	
	*Saussurea amara*	P	Sa	
	*Taraxacum mongolicum*	P	Tm	

LHT, life history traits; A, annual plants; P, perennial plants; AB, annual & biennial. +, plants were present in samples. The individual contribution values of coverage (%) of the most abundant species are listed. The abbreviations of the individual species are used in the RDA analyses.

The aboveground biomass levels were calculated using the linear regression model of the biomass-species richness relationship (species richness = a × biomass + b) as has been described in [38]. Ten plots located adjacent to the experimental plots were randomly selected. The species richness and biomass of every species in each plot were determined and then used to construct a regression equation (species richness = 0.040 × biomass + 7.34). The aboveground biomass of each experimental plot was then calculated using the regression equation.

The belowground biomass was estimated using the ingrowth core method. Two holes (7 cm diameter, 50 cm height) were drilled randomly in each plot using a soil drill. The collected soil samples were sieved to remove roots and then placed it into nylon mesh bags (the size of each bag was similar to the dimensions of the soil hole). Next, the nylon mesh bags were again carefully placed into the holes in the experiment plots. The nylon mesh bags were harvested on 18 July annually. The roots in each mesh bags were selected, washed, and then dried at 65°C for 48 h.

### Statistical analysis

All of the data analyses were performed using SPSS 16.0 (SPSS for Windows, Chicago, IL, USA), and all of the data were tested for normality of their distribution before performing statistical analyses. Four-way ANOVAs with a blocked nested design were performed to test for the main and interactive effects of block, year, warming and N addition on species composition (species diversity, richness, evenness and importance value) and biomass (aboveground and belowground biomass). A General Linear Model (GLM) with a Duncan test was used to examine the significant differences among treatments, and specific comparisons among treatments were performed using the LSMEANS statement. The constrained linear ordination technique of redundancy analysis (RDA) was performed using the PC-ORD 5 software to examine the response of community composition (in terms of species richness, diversity and density) to warming and N addition after four years of treatment (in 2009) according to the methods described by Yang et al. [5].

## Results

### Soil microclimate and available N content

**Fig 1:**
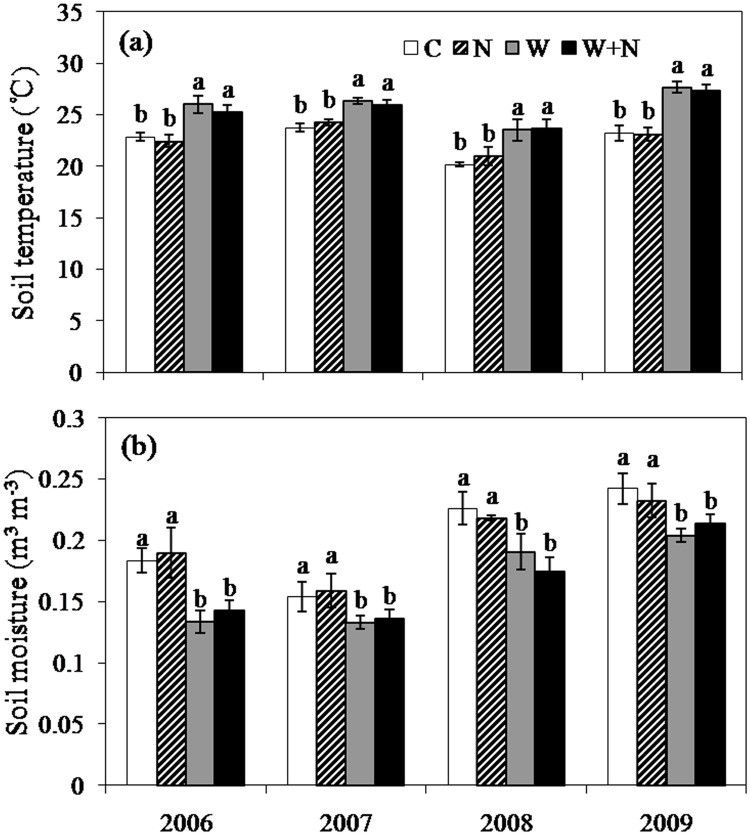
Effects of warming and N addition treatments on the seasonal and interannual variation in monthly surface layer (0–15 cm) soil mean temperature (a) and soil moisture (b) during the growing season from 2006 to 2009. Different lowercase letters above the columns indicate a significant difference (*P*<0.05) among treatments every year. Data are reported as the mean ± SE (n = 6).

There was strong interannual variability in precipitation, with the highest annual mean value (384 mm) in 2006 and the lowest annual mean value (217 mm) in 2007. The mean air temperature in 2008 was much lower than that in other years. Experimental warming had significant effects on soil temperature (ST) and soil moisture content (SM) across the 4 experimental years. Warming significantly elevated the ST (*P*<0.05). Compared to the control treatment, the mean annual ST in the warming plots and the warming plus N addition plots elevated by 1.71°C and 0.58°C, respectively; N addition had no indirect effect on soil temperature, e.g., through the shading of potentially better growing plants in these plots (*P*>0.05, Fig 1a). Warming reduced SM significantly (*P*<0.05), compared with the control treatment, experimental warming and warming plus N addition treatments caused reductions in the mean SM by 11.5% and 19.8%, respectively; whereas the impact of N addition on SM was not significant (*P*>0.05). Warming did not impact the soil available N content (*P*>0.05), but N addition increased the soil available N content to a great extent (*P*<0.05).

### Species richness, evenness and diversity

**Fig 2:**
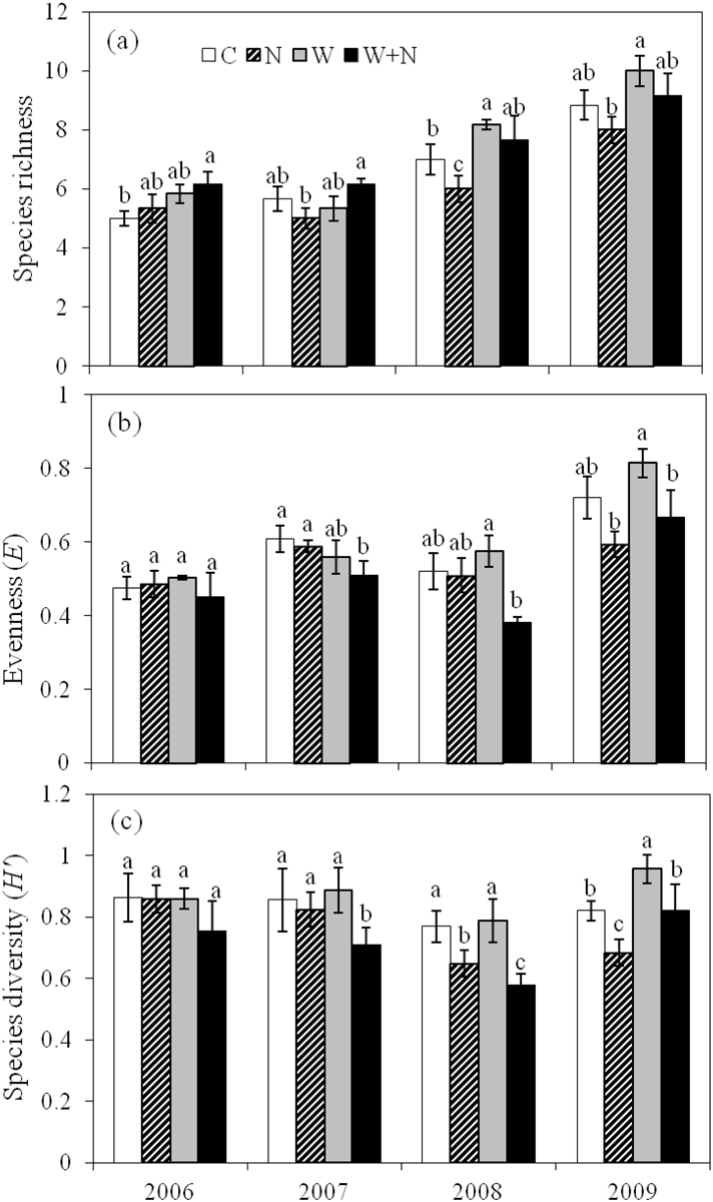
Effects of warming and N addition on plant species richness (a), evenness (b) and diversity (c) from 2006 to 2009. Different lowercase letters above the columns indicate a significant difference (*P*<0.05) among treatments every year. Data are reported as the mean ± SE (n = 6).

At the early stages of the experiment (in 2006), warming and N addition did not alter the species richness, evenness (Pielou index, *E*) or diversity (Shannon-Wiener index, *H*). With the progression of the warming and N addition treatments, the species richness, *E* and *H* values were altered significantly (Fig 2).

Warming enhanced species richness by an average of 11.6% (*P*<0.05) across the four experimental years (Fig 2a). In the N addition plots, species richness decreased on average by 15.8% (*P*<0.05) annually compared to the control plots from 2007 to 2009 (Fig 2a). There were no interactive effects of warming and N addition on species richness detected (*P =* 0.08). However, there were interactive effects of the experimental year and warming on species richness (*P<*0.05; Table 2). There was strong interannual variability in *E* (*P*<0.01), with the highest evenness (*E*) across all of the treatments in 2009 (0.71, Fig 2b). Neither the experimental warming (*P* = 0.09) nor the warming plus N addition (*P* = 0.055) treatments had a significant effect on *E* across the four experimental years.

**Table 2:** Results of four-way factorial ANOVA on the effects of year (Y), warming (W), N addition (N), and their interactions on the importance value of graminoid species (IVG), the importance value of forbs (IVF); richness (R); evenness (*E*); diversity (*H*); aboveground biomass (AB); belowground biomass (BB).

Source of variation	IVG	IVF	R	*E*	*H*	AB	BB
Block	**	**	*	**	*	**	**
Year	**	**	ns	*	**	**	**
W	ns	ns	ns	ns	ns	ns	ns
N	**	*	*	ns	**	**	**
Y×W	ns	ns	*	ns	ns	ns	ns
Y×N	ns	*	ns	ns	ns	**	ns
W×N	*	**	ns	ns	ns	ns	**
Y×W×N	ns	ns	ns	ns	ns	ns	ns

* *P*<0.05,

** *P*<0.01, “ns” indicates that the differences are not significant.

Warming enhanced species diversity (*H*) by 16.5% (*P*<0.05) in 2009; however, it did not affect *H* in other experimental years. The N addition treatment caused reductions in *H* by 15.8% (*P*<0.05) and 16.7% (*P*<0.05) in 2008 and 2009, respectively (Fig 2c). In the warming plus N addition treatment plots, the *H* decreased on average by 17.6% (*P*<0.05) compared to the control treatment across the four experimental years.

**Fig 3:**
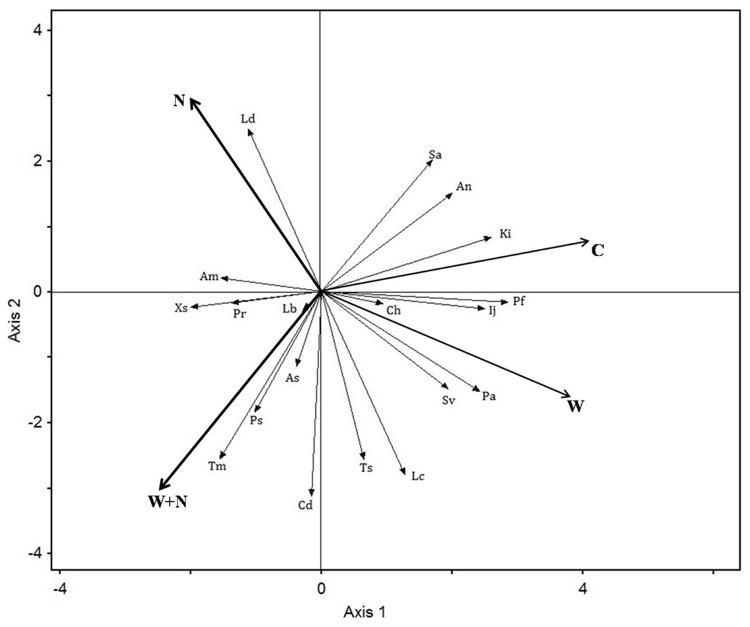
Ordination diagram showing the result of the redundancy analysis (RDA) of the plant community composition. C represents control; N represents N addition treatment; W represents warming treatment; W+N represents warming plus N addition treatment. See Table 1 for abbreviations of the species names.

The arrangement of the species in the ordination diagram (RDA, Fig 3) clearly reflects the divergence in the community composition according to the triangular symbols of the treatments (C, W, N and W+N). The warmed plots and the control plots had similar community compositions. The community compositions in the N addition treated plots were dissimilar from those in the warming and warming plus N addition treatments.

### Importance value

**Fig 4:**
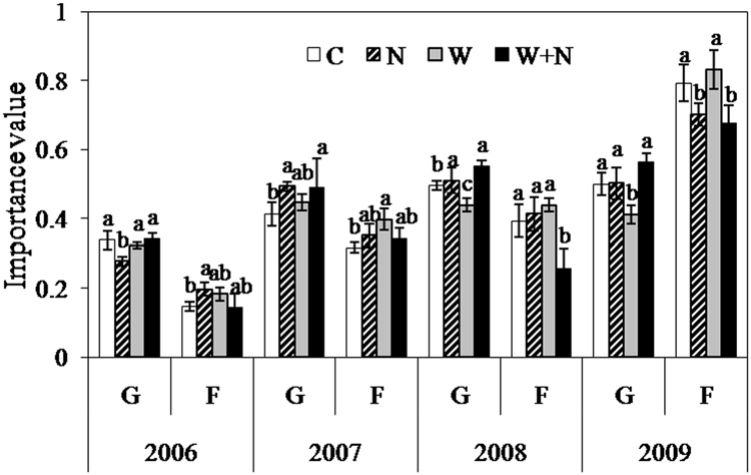
Effects of experimental warming and N addition on the importance values of graminoid species (G) and forbs (F). Different lowercase letters above the columns indicate significant differences (P<0.05) among treatments every year. Data are reported as the mean ± SE (n = 6).

During the four experimental years, the importance values (IVs) of the graminoids (*P*<0.01) and forbs (*P*<0.01) exhibited significant interannual variation (Table 2). Despite warming had no impact on the IVs of the graminoids in 2006 and 2007, warming caused reductions in the IVs by 11.8% (*P*<0.05) and 17.4% (*P*<0.05) in 2008 and 2009, respectively. The IV of the forbs in the warming plots increased by 13.6% (*P*<0.05) compared to the control treatment in 2007 (Fig 4). N addition significantly decreased the IV of the graminoids by 18.1% (*P*<0.01) in 2006 and enhanced it by 19.2% in 2007, but no impact on the IVs of the graminoids for 2008 and 2009. Compared to the control treatment, the IVs of the forbs in the N addition plots increased by 34.1% (*P*<0.05) and 11.1% in 2006 and 2007, respectively; however, the IV of the forbs decreased by 11.5% in 2009 (*P*<0.05). In the warming plus N addition treatment, the IV of the graminoid species increased by 11.5% (*P*<0.05) compared with the control treatment in 2008. The primary effects of the experimental years were N addition and the interactive effects of warming plus N addition on the IV of the graminoid species (*P*<0.01) (Table 2). The interactive effects of experimental year × N addition and warming × N addition on the IV of the forbs were also observed (*P*<0.05) (Table 2). The IV of the graminoid species was higher than that of the forbs across the four treatments from 2006 to 2008; however, the IV of the forbs was greater than that of the graminoids in 2009 (Fig 4).

### Aboveground and belowground biomass

**Fig 5:**
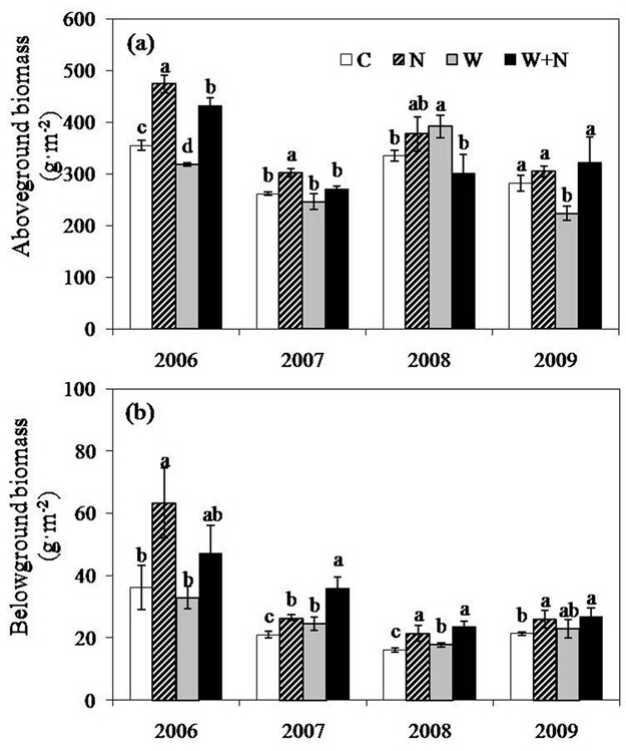
Effects of warming and nitrogen addition on aboveground biomass (a) and belowground biomass (b) during 2006 to 2009. Different lowercase letters above the columns indicate significant differences (P<0.05) among the treatments every year. Data are reported as the mean ± SE (n = 6).

Aboveground biomass exhibited an apparent interannual variation, with the highest (394.8 g m^-2^) and the lowest (270.2 g m^-2^) values occurring in 2006 and 2007, respectively (Fig 5a). Warming decreased the aboveground biomass by 9.2% (*P*<0.05) and 16.6% (*P*<0.05) in 2006 and 2009, respectively; however, warming increased the aboveground biomass by 20.8% (*P*<0.05) in 2008. On average, N addition increased the aboveground biomass by 20% (*P*<0.01) compared to the control plots from 2006 to 2009. Interactive effects of warming × N addition on the aboveground biomass (*P*<0.05) were only observed in 2006. Interactive effects of experimental year × N addition on the aboveground biomass (*P*<0.01) were also detected.

The mean belowground biomass across the four treatments in 2006 was much higher than those of the other three experimental years (Fig 5b). Warming treatment exhibited no effect on the belowground biomass in 2006; however, it increased the belowground biomass by 11.2% (*P*<0.05) in 2007, 2008 and 2009. N addition increased the belowground biomass by 6.1% (*P*<0.05) on average. From 2006 to 2009, the warming plus N addition treatment enhanced the belowground biomass by 50.7% (*P*<0.05) across the four experimental years. There were significant interactive effects of year × N addition and warming × N addition on the belowground biomass (Table 2).

## Discussion

### Effects of warming and N addition on soil N availability, temperature and moisture

Soil N availability is one of the most important limiting factors determining terrestrial ecosystem community composition and productivity [22,39]. Although there was no difference in soil N availability between the control and N addition treatments at the end of the growing season [29], N addition highly increased soil N availability during the growing season (May to October). Despite N availability was not increased by warming in the present study, N mineralization significantly increased in warmer conditions [29], which might be ascribed to warming increased activities of soil microorganism. As soil N availability is naturally low in the Songnen meadow steppe, an increase of soil N availability caused by N addition can affect the plant community composition and increase plant productivity, as reported in previous studies [22,39]. Moreover, N addition can reduce soil pH [40], which might affect the activities of the soil enzymes and indirectly influence the decomposition of organic matter, and thus increasing soil nutrient availability. Experimental warming significantly increased soil temperature and reduced soil moisture, whereas N addition did not affect soil temperature and moisture. These findings are in agreement with those of previous studies published on the same experiment [39,44].

### Effects of warming and N addition on plant community composition

In the present study, the plant community composition was altered significantly by four years of warming and N addition treatments. Across the four experimental years, the impact of warming on species richness was not significant (Table 2) but the richness tended to increase (Fig 2a) in the studied meadow steppe community, which is in accordance with the results observed for an annual grassland [6]. However, this result is inconsistent with the results of a number of other research studies in which warming has been associated with greater losses of species [5,41–43]. When analyzing the types of species growing in each treatment plot, we found that warming significantly increased the species number of the forbs (e.g., *Polygonum sibiricum* and *Taraxacum mongolicum*; Table 1), which might be related to the changes in soil moisture. Moreover, this result may be related to the changes in soil N mineralization caused by warming. In our previous studies, Ma et al. and Zhang et al. reported that warming increased the soil net N mineralization rate [29,44], which might be favor for the growth of annual forbs. The results suggest that short-lived forbs are more sensitive to changes of soil available N caused by global warming.

In contrast to the significant effects of warming on increasing species richness at the community level, N addition reduced the species richness in 2007 and 2008. This observed reduction in species richness under the N addition treatment is in agreement with the results obtained for a prairie grassland [16], a California annual grassland [6], European acidic grasslands [13–14], and a savannah grassland [45]. Previous studies have documented that the decline in grassland diversity in response to N addition is mainly determined by the loss of forbs [46]. We found that forb species significantly declined with N addition, which might explain why graminoid species may grow faster than forbs and indirectly suppress the growth of other groups through competition for light, nutrient resources, or water [47].

Current empirical and theoretical ecological results suggest that numerous species are at risk and that plant diversity will decline with the continuation of global warming [48]. A loss of plant diversity in association with experimental warming has been detected in numerous ecosystems [49]. However, a number of other studies have reported that plant diversity is not significantly affected by warming [5,43]. In our study, although warming did not affect plant diversity from 2006 to 2008, the diversity increased dramatically in the warming plots in 2009 (Fig 2). This observation can be explained on the basis of similar previous results that indicate that climate change indirectly affects the co-existing species by affecting the dominant species *L*. *chinensis* [50]. Moreover, the changes in the soil water availability caused by warming might be another factor that affects plant diversity. N addition had no impact on plant diversity during the first two experimental years but subsequently decreased plant diversity in 2008 and 2009. Our results are in agreement with the results of previous observations for a number of terrestrial ecosystems [6,51,52]. In the Songnen grassland soil, the total N (2 g kg^-1^) and the available N (40 mg kg^-1^) are very low [44], and while a small quantity or short-term N input cannot alter plant growth, but when soil N reach saturation for long-term or a large quantity N deposition the species diversity will decline. There was a positive linear relationship between plant diversity and productivity in a Eurasian steppe ecosystem [34]. The present results suggest that the reduction of plant diversity caused by N addition might reduce plant productivity in the future.

Over the four experimental years, the effects of warming and N addition on plant species richness and diversity increased gradually with the increase in N addition and extension of warming. These results suggest that long-term warming and N deposition might significantly alter plant community composition in our system and thus affect ecosystem function in the future. Furthermore, as the influences of warming and N deposition were affected by soil moisture; therefore, the effect of precipitation should be included in further studies on the effects of warming and N deposition on grassland ecosystems.

Changes in the IV of different functional groups can reflect the variation in interspecific relationships. We found that the IV of the graminoid species was considerably greater than that of the forbs from 2006 to 2008, but the IV of the forbs species was greater than that of the graminoid species in 2009. N addition highly increased the IV of the graminoid species in 2007, while warming decreased the IV in 2008 and 2009 and increased the IV of the forbs in 2007 (Fig 4). The significant response of the IV of the graminoid (*P*<0.01) and forb species (*P*<0.05) to N addition might be related to the different response of individuals, which then can alter plant interspecific competition ability. Furthermore, significant variations in plant species responses to warming and N addition were observed in our system (Fig 3), contributing to shifts in the competitive abilities of these species and the community composition [48,53]. These results may be partially ascribed to the changes in interspecific competition, suggesting that climate change can indirectly affect subdominant species via the dominant species. These observations highlight the potential of climate changes to alter species interactions.

Furthermore, we found that the warming in the N addition plots affected species richness and diversity in a different manner than in the ambient N plots. The plant species richness and diversity in the warming plus N addition plots were substantially lower than in the warming plots. These results can be ascribed to the stimulation of the graminoid species and the inhibition of the forbs caused by warming and N addition (Fig 4); hence the interactive effects of warming × N addition on the IVs of graminoid species and forbs play a key role in determining plant community composition (Table 2). These results also suggest that warming might, to a certain extent, reduce the negative effects of N addition on plant communities. This result might occur because warming can improve the activities of soil enzymes and increase the soil microbial biomass [54]. Increased soil enzyme activity can indirectly influence the plant community by altering the nutrient availability for plants in soils [55].

### Effects of warming and N addition on plant community biomass production

Warming tended to reduce the aboveground biomass in the Songnen meadow ecosystem, except in 2008. Our observations are in accordance with the results of several studies conducted on annual grasslands [6], an old field site [56], and in the whole of Europe [11]. The effect of warming varied among the years, which might be related to the fluctuations in the precipitation and in the atmospheric temperature. In 2008, the mean atmospheric temperature was lower than in other years, whereas the amount of precipitation was greater. We suggest that the lower atmospheric temperature and higher rainfall decreased the negative effect of experimental warming on the aboveground biomass [31]. There were no significant effects observed warming on the belowground biomass across the four experimental years, which is consistent with the results found by Asner et al. [57]. However, the belowground biomass tended to increase under the warming treatment, especially in 2007 and 2008, which is in line with previous results reported for North America [27], suggesting that global warming might stimulate the growth of the root system and alter the carbon allocation between the aboveground and belowground biomass components.

Nitrogen is one of the most essential elements for the development of plant species, and the degree of N limitation often determines terrestrial ecosystem net primary productivity [22,27]. Our results demonstrate that N addition significantly increased the aboveground and belowground biomass, which is consistent with the results of previous studies [26–27] but inconsistent with the results observed for the Wasatch Plateau [58]. Plant species can respond rapidly to changes in soil N availability [8]; when the soil available N increased, the growth of plants greatly improved, as evidence by the increase in total biomass production in this area. However, in the present study, we found that the aboveground biomass exhibited a tendency to decline under N addition, which might be ascribed to the decrease in plant species richness and diversity caused by N addition. Furthermore, the effect of N deposition on plant productivity might be influenced by soil moisture. Model simulation results suggest that N addition increases ecosystem productivity when soil moisture is higher rather than lower in a semiarid ecosystem [51]. In fact, we observed that the effects of N addition on the aboveground biomass in the wet years (in 2006 and 2008) were substantially greater than in the drought years (in 2007 and 2009).

In addition, significant differences were observed between the warming and warming plus N addition treatments in the aboveground and belowground biomass levels. This result further demonstrates that N is an important limiting factor for plant productivity in the Songnen meadow steppe; furthermore, the improvement in plant growth attributed to increased N deposition might alleviate the negative effects of warming on plant growth.

### Summary

Our study has revealed that both warming and N addition altered both plant community composition and biomass production in the Songnen meadow ecosystem. Warming had no significant impact on species richness. N addition highly decreased species richness and diversity. This effect is gradually enhanced over time with N enrichment. Warming had no significant influence on the aboveground biomass but increased the belowground productivity. N addition highly enhanced both the aboveground and the belowground productivity. The synergetic effect of warming and N addition on the belowground productivity was also detected in the later three experimental years. These observations have further improved our understanding of how community composition and biomass production in temperate meadow ecosystems respond to these simultaneous climate changes.

## Supporting Information

S1 Fig Average monthly air temperature and daily precipitation during Aug. 2006 to Aug. 2009. Data was from the eddy tower adjacent (approximately 200 m) to the experimental plots.
(TIF)

S2 Fig Effects of warming and nitrogen addition on soil available N concentration during 2006 to 2009. Different lowercase letters on columns indicate significant difference (*P*<0.05) among treatments every year. Data are reported as means ± SE (n = 6).
(TIF)
