# Supplementary figures and images for: Responses of Plant Community Composition and Biomass Production to Warming and Nitrogen Deposition in a Temperate Meadow Ecosystem
Source: PLoS One. 2015 Apr 13;10(4):e0123160. doi: 10.1371/journal.pone.0123160 (PMC4395313; doi:10.1371/journal.pone.0123160)

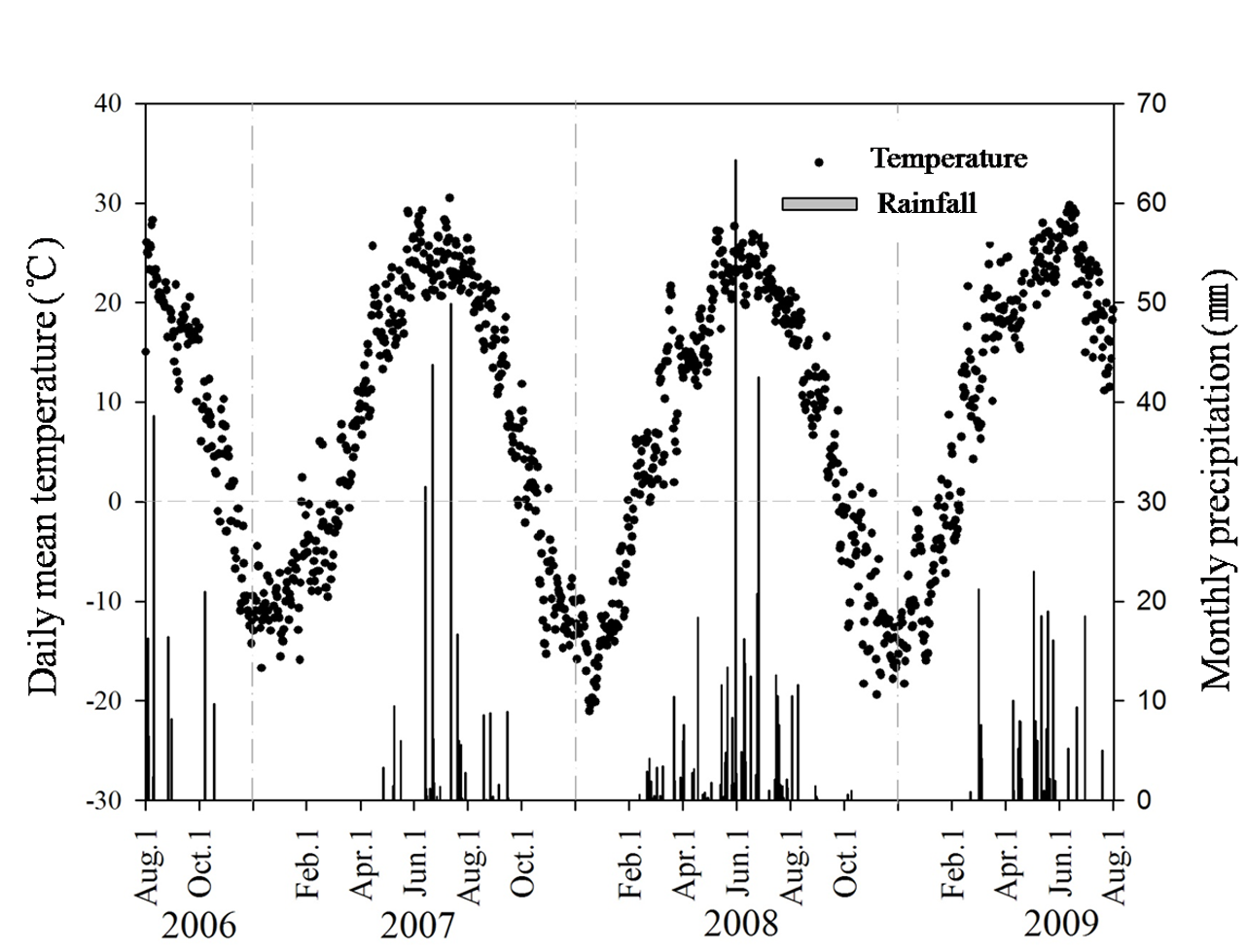

Supplement: S1 Fig — Data was from the eddy tower adjacent (approximately 200 m) to the experimental plots. (TIF) [file pone.0123160.s001.tif]

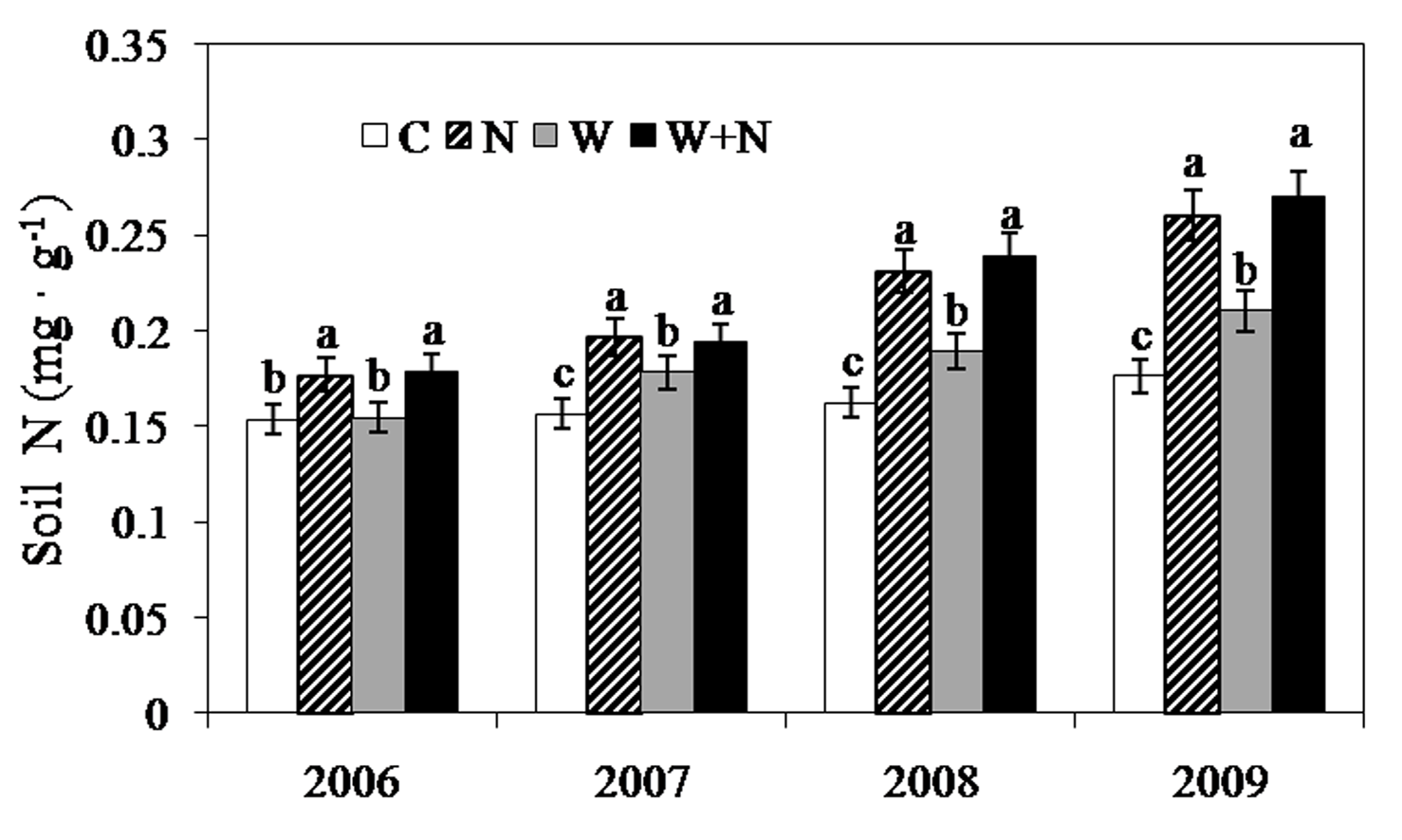

Supplement: S2 Fig — Different lowercase letters on columns indicate significant difference (P<0.05) among treatments every year. Data are reported as means ± SE (n = 6). (TIF) [file pone.0123160.s002.tif]
